# [5-Chloro-2-hy­droxy-*N*′-(2-oxidobenzyl­idene)benzohydrazidato]dimethyl­tin(IV)

**DOI:** 10.1107/S1600536811025621

**Published:** 2011-07-06

**Authors:** Xiuyun Zhang, Caihong Yue, Handong Yin

**Affiliations:** a College of Chemistry and Chemical Engineering, Liaocheng University, Shandong 252059, People’s Republic of China

## Abstract

In the title compound, [Sn(CH_3_)_2_(C_14_H_9_ClN_2_O_3_)], the Sn^IV^ ion is coordinated by one N and two O atoms from the tridentate 5-chloro-2-hy­droxy-*N*′-(2-oxidobenzyl­idene)benzohydrazidate (*L*) ligand and two methyl groups in a distorted trigonal–bipyramidal geometry. In the ligand, the hy­droxy group is involved in an intra­molecular O—H⋯N hydrogen bond and the two aromatic rings form a dihedral angle of 5.5 (1)°. In the crystal, weak inter­molecular C—H⋯O hydrogen bonds and π–π inter­actions between the aromatic rings [centroid–centroid distance = 3.816 (3) Å] link the mol­ecules into centrosymmetric dimers.

## Related literature

For related structures, see: Yearwood *et al.* (2002 ▶); Hong *et al.* (2010 ▶); Li *et al.* (2009 ▶).
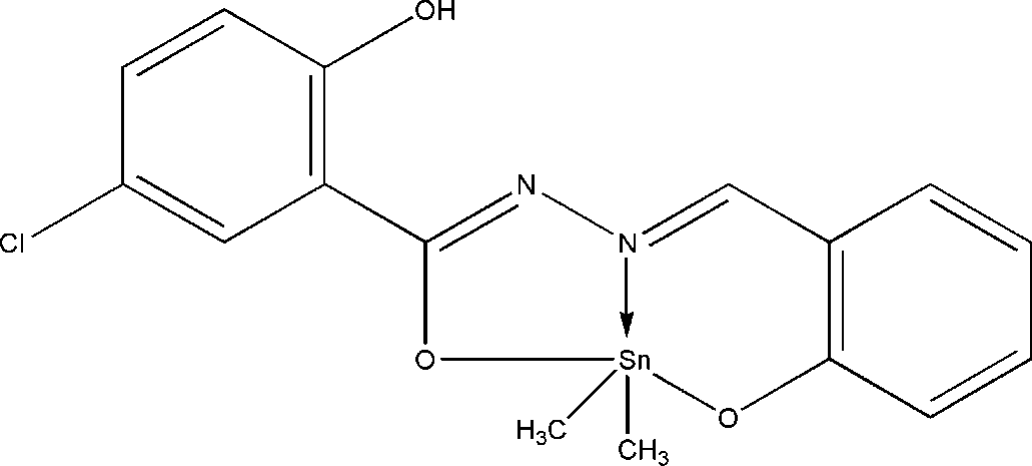


## Experimental

### 

#### Crystal data

[Sn(CH_3_)_2_(C_14_H_9_ClN_2_O_3_)]
*M* *_r_* = 437.44
Monoclinic, 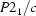

*a* = 7.5096 (5) Å
*b* = 20.965 (2) Å
*c* = 10.8344 (11) Å
β = 95.634 (1)°
*V* = 1697.5 (3) Å^3^
*Z* = 4
Mo *K*α radiation
μ = 1.68 mm^−1^
*T* = 298 K
0.48 × 0.41 × 0.23 mm

#### Data collection

Bruker SMART CCD area-detector diffractometer
Absorption correction: multi-scan (*SADABS*; Bruker, 2001 ▶) *T*_min_ = 0.500, *T*_max_ = 0.699
8414 measured reflections
2975 independent reflections
2357 reflections with *I* > 2σ(*I*)
*R*_int_ = 0.052

#### Refinement

*R*[*F* ^2^ > 2σ(*F* ^2^)] = 0.029
*wR*(*F* ^2^) = 0.071
*S* = 1.07
2975 reflections
212 parameters
H-atom parameters constrained
Δρ_max_ = 0.44 e Å^−3^
Δρ_min_ = −0.67 e Å^−3^

### 

Data collection: *SMART* (Bruker, 2007 ▶); cell refinement: *SAINT* (Bruker, 2007 ▶); data reduction: *SAINT*; program(s) used to solve structure: *SHELXS97* (Sheldrick, 2008 ▶); program(s) used to refine structure: *SHELXL97* (Sheldrick, 2008 ▶); molecular graphics: *SHELXTL* (Sheldrick, 2008 ▶); software used to prepare material for publication: *SHELXTL*.

## Supplementary Material

Crystal structure: contains datablock(s) I, global. DOI: 10.1107/S1600536811025621/cv5115sup1.cif

Structure factors: contains datablock(s) I. DOI: 10.1107/S1600536811025621/cv5115Isup2.hkl

Additional supplementary materials:  crystallographic information; 3D view; checkCIF report

## Figures and Tables

**Table 1:** Hydrogen-bond geometry (Å, °)

*D*—H⋯*A*	*D*—H	H⋯*A*	*D*⋯*A*	*D*—H⋯*A*
C15—H15*A*⋯O1^i^	0.96	2.59	3.430 (5)	147
O1—H1⋯N1	0.82	1.87	2.577 (4)	144

Symmetry code: (i) 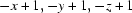.

**Fig. 1.**
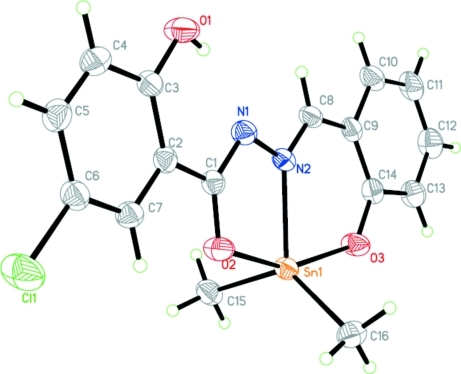
The molecular structure of (I) showing the atomic numbering and 50% probability displacement ellipsoids.

**Fig. 2.**
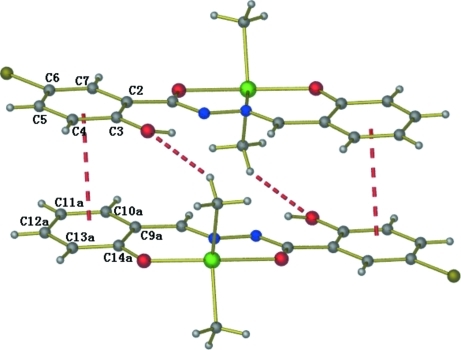
Centrosymmetric dimer in the crystal structure of (I) [symmetry code: (a) - x + 1,-y + 1,-z + 1]. Dashed lines denote intermolecular C—H···O hydrogen bonds and π—π interactions.

## supplementary crystallographic information

### Comment

Organotin(IV) compounds of hydrazone Schiff bases were reported by Hong *et al.* (2010). In continuation of our study of hydrazone Schiff base organotin(IV) compounds (Li *et al.*, 2009), we have synthesized the title compound,  (I).

In (I) (Fig. 1), the tin center is five-coordinated in a distorted trigonal bipyramidal geometry, being surrounded by two C atoms from the alkyl and one N atom, two O atoms from the Schiff base ligand. So the ligand coordinated to the tin atom as a tridentate ligand. In the tridentate ligand, two aromatic rings form a dihedral angle of 5.5 (1)°. The O atoms coordinate the Sn center with different bond lengths - for carbonyl O2 atom Sn—O2 = 2.183 (2) Å, and for hydroxy O3 atom Sn—O3 = 2.078 (3) Å. Similar structural parameters were observed in the related compound (Yearwood *et al.*, 2002). In (I), the angles o C15–Sn–C16, C15–Sn–O3 and C16–Sn–O3 are 129.30 (15)°, 100.22 (14)° and 95.08 (14)°, respectively, indicating the distorted trigonal bipyramidal coordination geometry.

In the crystal structure, weak intermolecular C—H···O hydrogen bonds (Table 1) and π—π interactions between the aromatic rings [centroid-to-centroid distance of 3.816 (3) Å] link the molecules into centrosymmetric dimers (Fig.2 ).

### Experimental

The reaction was carried out under nitrogen atmosphere. The Schiff base ligand (0.2 mmol) was added to 30 ml e thanol with sodium ethoxide (0.4 mmol). The mixture was stirred for 0.5 h and then dichlorodimethyltin (0.2 mmol) was added.And the mixture was stirred for 12 h under reflux. After cooling to room temperature, the mixture was filtered and evaporated to dryness. The resulting solid, was then recrystallized from dichloromethane-petroleum ether (1:1, *v*/*v*). Anal. Calcd (%) for C_16_H_15_Cl_N_2_O_3_Sn_ (Mr = 437.44): C, 43.93; H, 3.46; N, 6.40; O, 10.97. Found (%): C, 43.90; H, 3.42; N, 6.35; O, 10.9.

### Refinement

The H atoms were positioned geometrically (C–H 0.93-0.96 Å; O–H 0.82 Å), and refined as riding, with *U*_iso_(H) = 1.2-1.5 *U*_eq_ of the parent atom.

### Crystal data

**Table:**

[Sn(CH_3_)_2_(C_14_H_9_ClN_2_O_3_)]	*F*(000) = 864
*M**_r_* = 437.44	*D*_x_ = 1.712 Mg m^−^^3^
Monoclinic, *P*2_1_/*c*	Mo *K*α radiation, λ = 0.71073 Å
*a* = 7.5096 (5) Å	Cell parameters from 4020 reflections
*b* = 20.965 (2) Å	θ = 3.3–27.2°
*c* = 10.8344 (11) Å	µ = 1.68 mm^−^^1^
β = 95.634 (1)°	*T* = 298 K
*V* = 1697.5 (3) Å^3^	Block, yellow
*Z* = 4	0.48 × 0.41 × 0.23 mm

### Data collection

**Table:**

Bruker SMART CCD area-detector diffractometer	2975 independent reflections
Radiation source: fine-focus sealed tube	2357 reflections with *I* > 2σ(*I*)
graphite	*R*_int_ = 0.052
φ and ω scans	θ_max_ = 25.0°, θ_min_ = 2.7°
Absorption correction: multi-scan (*SADABS*; Bruker, 2001)	*h* = −8→8
*T*_min_ = 0.500, *T*_max_ = 0.699	*k* = −24→23
8414 measured reflections	*l* = −12→12

### Refinement

**Table:**

Refinement on *F*^2^	Secondary atom site location: difference Fourier map
Least-squares matrix: full	Hydrogen site location: inferred from neighbouring sites
*R*[*F*^2^ > 2σ(*F*^2^)] = 0.029	H-atom parameters constrained
*wR*(*F*^2^) = 0.071	*w* = 1/[σ^2^(*F*_o_^2^) + (0.026*P*)^2^ + 0.2305*P*] where *P* = (*F*_o_^2^ + 2*F*_c_^2^)/3
*S* = 1.07	(Δ/σ)_max_ = 0.001
2975 reflections	Δρ_max_ = 0.44 e Å^−^^3^
212 parameters	Δρ_min_ = −0.67 e Å^−^^3^
0 restraints	Extinction correction: *SHELXL97* (Sheldrick, 2008), Fc^*^=kFc[1+0.001xFc^2^λ^3^/sin(2θ)]^-1/4^
Primary atom site location: structure-invariant direct methods	Extinction coefficient: 0.0182 (6)

### Fractional atomic coordinates and isotropic or equivalent isotropic displacement parameters (Å^2^)

**Table:**

	*x*	*y*	*z*	*U*_iso_*/*U*_eq_	
Sn1	0.36782 (3)	0.466514 (11)	0.80395 (2)	0.03921 (13)	
Cl1	−0.1037 (2)	0.21038 (5)	0.43753 (11)	0.0807 (4)	
N1	0.2415 (4)	0.48410 (14)	0.5277 (3)	0.0413 (7)	
N2	0.3277 (4)	0.51754 (13)	0.6279 (3)	0.0365 (7)	
O1	0.1173 (5)	0.46606 (13)	0.3001 (2)	0.0606 (8)	
H1	0.1802	0.4835	0.3564	0.091*	
O2	0.2336 (4)	0.40168 (12)	0.6672 (2)	0.0498 (7)	
O3	0.4514 (4)	0.55533 (12)	0.8723 (2)	0.0534 (7)	
C1	0.1973 (5)	0.42608 (17)	0.5585 (3)	0.0369 (8)	
C2	0.1016 (5)	0.38655 (16)	0.4607 (3)	0.0372 (8)	
C3	0.0701 (5)	0.40754 (18)	0.3373 (3)	0.0413 (9)	
C4	−0.0106 (5)	0.36639 (19)	0.2477 (3)	0.0501 (10)	
H4	−0.0283	0.3799	0.1656	0.060*	
C5	−0.0637 (6)	0.30721 (19)	0.2777 (3)	0.0492 (10)	
H5	−0.1184	0.2805	0.2168	0.059*	
C6	−0.0362 (5)	0.28668 (17)	0.3994 (3)	0.0455 (9)	
C7	0.0456 (5)	0.32594 (17)	0.4898 (3)	0.0407 (9)	
H7	0.0635	0.3116	0.5713	0.049*	
C8	0.3792 (5)	0.57425 (17)	0.6034 (3)	0.0409 (9)	
H8	0.3597	0.5870	0.5210	0.049*	
C9	0.4634 (5)	0.62022 (16)	0.6886 (3)	0.0405 (9)	
C10	0.5084 (5)	0.67917 (18)	0.6401 (4)	0.0532 (10)	
H10	0.4906	0.6858	0.5549	0.064*	
C11	0.5786 (6)	0.72764 (19)	0.7158 (4)	0.0607 (12)	
H11	0.6094	0.7666	0.6826	0.073*	
C12	0.6020 (6)	0.7170 (2)	0.8418 (4)	0.0611 (12)	
H12	0.6478	0.7497	0.8936	0.073*	
C13	0.5603 (6)	0.66031 (19)	0.8930 (4)	0.0554 (11)	
H13	0.5786	0.6551	0.9785	0.066*	
C14	0.4897 (5)	0.60941 (18)	0.8185 (3)	0.0444 (9)	
C15	0.6154 (5)	0.41890 (19)	0.8108 (4)	0.0536 (10)	
H15A	0.6874	0.4378	0.7521	0.080*	
H15B	0.5956	0.3747	0.7907	0.080*	
H15C	0.6761	0.4224	0.8927	0.080*	
C16	0.1613 (6)	0.45669 (19)	0.9204 (4)	0.0528 (11)	
H16A	0.2094	0.4617	1.0052	0.079*	
H16B	0.1083	0.4152	0.9091	0.079*	
H16C	0.0719	0.4887	0.8999	0.079*	

### Atomic displacement parameters (Å^2^)

**Table:**

	*U*^11^	*U*^22^	*U*^33^	*U*^12^	*U*^13^	*U*^23^
Sn1	0.0436 (2)	0.03337 (18)	0.04025 (17)	−0.00176 (11)	0.00207 (11)	0.00346 (11)
Cl1	0.1181 (11)	0.0464 (7)	0.0724 (8)	−0.0281 (7)	−0.0178 (7)	0.0042 (6)
N1	0.049 (2)	0.0347 (17)	0.0402 (16)	−0.0027 (14)	0.0022 (14)	0.0004 (14)
N2	0.0372 (18)	0.0300 (16)	0.0424 (16)	0.0005 (13)	0.0037 (13)	0.0041 (13)
O1	0.087 (2)	0.0491 (18)	0.0438 (16)	−0.0106 (15)	−0.0039 (15)	0.0111 (13)
O2	0.0696 (19)	0.0399 (15)	0.0378 (14)	−0.0116 (14)	−0.0056 (13)	0.0044 (12)
O3	0.078 (2)	0.0340 (14)	0.0468 (15)	−0.0133 (14)	0.0014 (14)	0.0037 (13)
C1	0.035 (2)	0.037 (2)	0.040 (2)	0.0052 (16)	0.0058 (16)	0.0016 (16)
C2	0.036 (2)	0.038 (2)	0.0373 (19)	0.0063 (16)	0.0004 (15)	−0.0022 (16)
C3	0.045 (2)	0.036 (2)	0.043 (2)	0.0046 (17)	0.0081 (17)	0.0042 (17)
C4	0.057 (3)	0.054 (3)	0.038 (2)	0.000 (2)	−0.0014 (18)	−0.0012 (19)
C5	0.053 (3)	0.050 (2)	0.044 (2)	0.001 (2)	−0.0006 (18)	−0.0085 (19)
C6	0.050 (3)	0.038 (2)	0.047 (2)	−0.0004 (18)	0.0005 (18)	−0.0006 (17)
C7	0.043 (2)	0.042 (2)	0.0366 (19)	0.0005 (17)	−0.0003 (16)	0.0010 (17)
C8	0.042 (2)	0.036 (2)	0.047 (2)	0.0040 (17)	0.0100 (17)	0.0106 (17)
C9	0.039 (2)	0.030 (2)	0.054 (2)	0.0014 (16)	0.0118 (18)	0.0011 (17)
C10	0.056 (3)	0.038 (2)	0.066 (3)	0.0014 (19)	0.010 (2)	0.008 (2)
C11	0.063 (3)	0.033 (2)	0.089 (3)	−0.007 (2)	0.020 (3)	0.001 (2)
C12	0.058 (3)	0.044 (3)	0.082 (3)	−0.011 (2)	0.009 (2)	−0.011 (2)
C13	0.064 (3)	0.041 (2)	0.061 (3)	−0.007 (2)	0.004 (2)	−0.008 (2)
C14	0.040 (2)	0.038 (2)	0.056 (2)	0.0007 (18)	0.0088 (18)	−0.0022 (19)
C15	0.050 (3)	0.047 (2)	0.064 (3)	0.009 (2)	0.007 (2)	0.010 (2)
C16	0.052 (3)	0.062 (3)	0.045 (2)	0.001 (2)	0.0056 (19)	0.0049 (19)

### Geometric parameters (Å, °)

**Table:**

Sn1—O3	2.078 (3)	C6—C7	1.378 (5)
Sn1—C16	2.103 (4)	C7—H7	0.9300
Sn1—C15	2.105 (4)	C8—C9	1.437 (5)
Sn1—N2	2.182 (3)	C8—H8	0.9300
Sn1—O2	2.183 (2)	C9—C10	1.398 (5)
Cl1—C6	1.740 (4)	C9—C14	1.420 (5)
N1—C1	1.312 (4)	C10—C11	1.377 (6)
N1—N2	1.397 (4)	C10—H10	0.9300
N2—C8	1.286 (4)	C11—C12	1.377 (6)
O1—C3	1.349 (4)	C11—H11	0.9300
O1—H1	0.8200	C12—C13	1.362 (6)
O2—C1	1.288 (4)	C12—H12	0.9300
O3—C14	1.319 (4)	C13—C14	1.409 (5)
C1—C2	1.476 (5)	C13—H13	0.9300
C2—C7	1.385 (5)	C15—H15A	0.9600
C2—C3	1.405 (5)	C15—H15B	0.9600
C3—C4	1.393 (5)	C15—H15C	0.9600
C4—C5	1.353 (5)	C16—H16A	0.9600
C4—H4	0.9300	C16—H16B	0.9600
C5—C6	1.383 (5)	C16—H16C	0.9600
C5—H5	0.9300	CG1—CG2^i^	3.8154 (2)
			
O3—Sn1—C16	95.08 (14)	C6—C7—H7	119.7
O3—Sn1—C15	100.23 (14)	C2—C7—H7	119.7
C16—Sn1—C15	129.29 (15)	N2—C8—C9	127.6 (3)
O3—Sn1—N2	83.30 (10)	N2—C8—H8	116.2
C16—Sn1—N2	121.70 (13)	C9—C8—H8	116.2
C15—Sn1—N2	108.00 (13)	C10—C9—C14	119.9 (3)
O3—Sn1—O2	154.56 (10)	C10—C9—C8	117.3 (3)
C16—Sn1—O2	91.59 (13)	C14—C9—C8	122.6 (3)
C15—Sn1—O2	94.35 (13)	C11—C10—C9	121.4 (4)
N2—Sn1—O2	72.36 (10)	C11—C10—H10	119.3
C1—N1—N2	112.1 (3)	C9—C10—H10	119.3
C8—N2—N1	115.4 (3)	C12—C11—C10	118.4 (4)
C8—N2—Sn1	128.0 (2)	C12—C11—H11	120.8
N1—N2—Sn1	116.6 (2)	C10—C11—H11	120.8
C3—O1—H1	109.5	C13—C12—C11	122.2 (4)
C1—O2—Sn1	114.5 (2)	C13—C12—H12	118.9
C14—O3—Sn1	133.1 (2)	C11—C12—H12	118.9
O2—C1—N1	124.4 (3)	C12—C13—C14	121.1 (4)
O2—C1—C2	118.6 (3)	C12—C13—H13	119.4
N1—C1—C2	117.1 (3)	C14—C13—H13	119.4
C7—C2—C3	118.6 (3)	O3—C14—C13	118.9 (3)
C7—C2—C1	119.3 (3)	O3—C14—C9	124.0 (3)
C3—C2—C1	122.1 (3)	C13—C14—C9	117.0 (4)
O1—C3—C4	117.7 (3)	Sn1—C15—H15A	109.5
O1—C3—C2	123.0 (3)	Sn1—C15—H15B	109.5
C4—C3—C2	119.4 (4)	H15A—C15—H15B	109.5
C5—C4—C3	121.3 (4)	Sn1—C15—H15C	109.5
C5—C4—H4	119.4	H15A—C15—H15C	109.5
C3—C4—H4	119.4	H15B—C15—H15C	109.5
C4—C5—C6	119.6 (4)	Sn1—C16—H16A	109.5
C4—C5—H5	120.2	Sn1—C16—H16B	109.5
C6—C5—H5	120.2	H16A—C16—H16B	109.5
C7—C6—C5	120.4 (4)	Sn1—C16—H16C	109.5
C7—C6—Cl1	120.0 (3)	H16A—C16—H16C	109.5
C5—C6—Cl1	119.5 (3)	H16B—C16—H16C	109.5
C6—C7—C2	120.7 (3)		
			
C1—N1—N2—C8	178.1 (3)	C7—C2—C3—C4	−2.1 (5)
C1—N1—N2—Sn1	0.5 (4)	C1—C2—C3—C4	175.8 (3)
O3—Sn1—N2—C8	10.7 (3)	O1—C3—C4—C5	−179.2 (4)
C16—Sn1—N2—C8	102.5 (3)	C2—C3—C4—C5	1.8 (6)
C15—Sn1—N2—C8	−87.9 (3)	C3—C4—C5—C6	−0.6 (6)
O2—Sn1—N2—C8	−176.8 (3)	C4—C5—C6—C7	−0.4 (6)
O3—Sn1—N2—N1	−172.0 (2)	C4—C5—C6—Cl1	−179.7 (3)
C16—Sn1—N2—N1	−80.1 (3)	C5—C6—C7—C2	0.1 (6)
C15—Sn1—N2—N1	89.4 (3)	Cl1—C6—C7—C2	179.4 (3)
O2—Sn1—N2—N1	0.5 (2)	C3—C2—C7—C6	1.2 (5)
O3—Sn1—O2—C1	16.0 (4)	C1—C2—C7—C6	−176.8 (3)
C16—Sn1—O2—C1	121.4 (3)	N1—N2—C8—C9	177.0 (3)
C15—Sn1—O2—C1	−109.0 (3)	Sn1—N2—C8—C9	−5.6 (5)
N2—Sn1—O2—C1	−1.5 (2)	N2—C8—C9—C10	179.8 (4)
C16—Sn1—O3—C14	−132.8 (4)	N2—C8—C9—C14	−4.8 (6)
C15—Sn1—O3—C14	95.7 (4)	C14—C9—C10—C11	0.1 (6)
N2—Sn1—O3—C14	−11.5 (4)	C8—C9—C10—C11	175.6 (4)
O2—Sn1—O3—C14	−28.3 (5)	C9—C10—C11—C12	−0.7 (6)
Sn1—O2—C1—N1	2.6 (4)	C10—C11—C12—C13	0.9 (7)
Sn1—O2—C1—C2	−178.2 (2)	C11—C12—C13—C14	−0.3 (7)
N2—N1—C1—O2	−2.1 (5)	Sn1—O3—C14—C13	−174.0 (3)
N2—N1—C1—C2	178.7 (3)	Sn1—O3—C14—C9	6.4 (6)
O2—C1—C2—C7	3.3 (5)	C12—C13—C14—O3	180.0 (4)
N1—C1—C2—C7	−177.4 (3)	C12—C13—C14—C9	−0.4 (6)
O2—C1—C2—C3	−174.6 (3)	C10—C9—C14—O3	−179.9 (4)
N1—C1—C2—C3	4.8 (5)	C8—C9—C14—O3	4.8 (6)
C7—C2—C3—O1	179.0 (3)	C10—C9—C14—C13	0.5 (5)
C1—C2—C3—O1	−3.1 (5)	C8—C9—C14—C13	−174.8 (3)

Symmetry codes: (i) −*x*+1, −*y*+1, −*z*+1.

### Hydrogen-bond geometry (Å, °)

**Table:**

*D*—H···*A*	*D*—H	H···*A*	*D*···*A*	*D*—H···*A*
C15—H15A···O1^i^	0.96	2.59	3.430 (5)	147
O1—H1···N1	0.82	1.87	2.577 (4)	144

Symmetry codes: (i) −*x*+1, −*y*+1, −*z*+1.
